# An Axonal Growth Pathway Requires an Alzheimer's Protein

**DOI:** 10.1371/journal.pbio.1001559

**Published:** 2013-05-14

**Authors:** Richard Robinson

**Affiliations:** Freelance Science Writer, Sherborn, Massachusetts, United States of America

The brain of a patient with Alzheimer's disease is clogged with plaques—clumps of protein whose main constituent is a small polypeptide called amyloid-beta, or a-beta. A-beta is cleaved from a larger protein called amyloid precursor protein (APP). A drug to clear a-beta remains at the top of the (unfulfilled) wish list for the disease, but it remains unclear whether the clumps are the cause or consequence of the disease, and thus whether a-beta, or some other entity, is the best target for therapy. That uncertainty is magnified by uncertainty about the function of APP—despite years of research into the pathophysiology of Alzheimer's disease, there is still no clear understanding of what APP does outside the context of the disease. In this issue of *PLOS Biology*, Alessia Soldano, Bassem Hassan, and colleagues show that in the developing brain, the fly homolog of APP is needed for normal axonal outgrowth, and acts through a highly conserved signaling pathway.

**Figure:**
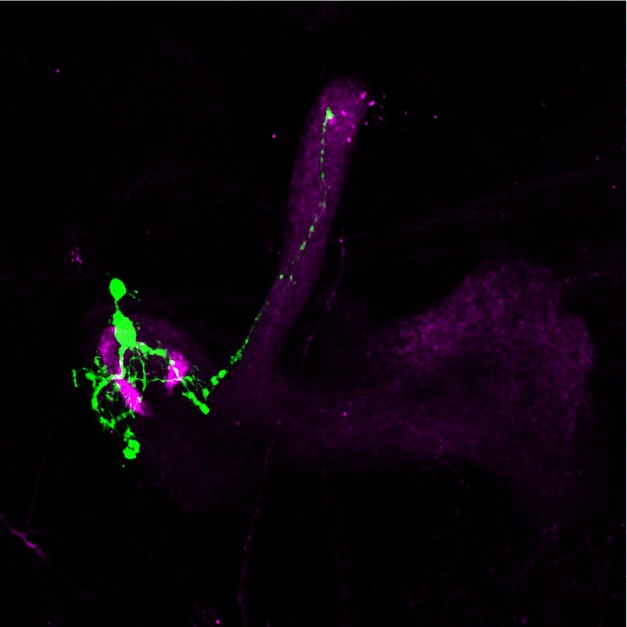
A clone of APPL mutant mushroom body neurons marked with GFP (green), in a background of wild type mushroom body lobes (magenta), showing failure of the mutant axons to grow into the medial, or beta, lobe.

The fly version of APP, called APPL, is expressed throughout the brain, but especially in the so-called alpha-beta neurons of the mushroom bodies, critical for learning and memory. The axon of an alpha-beta neuron splits into alpha and beta branches; branches from multiple neurons cluster with others of their kind to form alpha and beta lobes. When the authors deleted APPL, 26% of the flies developed axonal defects: about half of them with poorly developed alpha lobes, half with defective beta lobes. That partial disruption of development suggested that APPL in some way facilitated, but did not strictly control, outgrowth. When they deleted one copy of an enzyme called Abelson kinase (Abl), also known to be involved in axonal outgrowth, that 26% became 51%; conversely, overexpression of the kinase compensated for APPL loss, suggesting the two acted within the same pathway.

Work by other groups has shown that the target for Abl is a protein called Disheveled, a key player in the Wnt-PCP signaling pathway. In epithelial cells, this pathway determines cell polarity, and in the nervous system regulates axonal outgrowth, relying on interactions among a group of colorfully named proteins, including Frizzled, Flamingo, and VanGogh, along with Disheveled. Disabling Disheveled's kinase site interrupted development of the beta lobes; loss of either Frizzled or VanGogh had a similar effect. In all cases, the effect was partial, again suggesting that each contributed to some larger operational entity.

Overexpression of Disheveled largely reversed the effects of APPL loss, strengthening the case that APPL's role is, in combination with the other members of the group, to bring together the kinase and its substrate to promote efficient signaling.

Turning to mice, whose APP resembles the human version, the authors showed that phosphorylation of the mouse version of Disheveled was disrupted by loss of APP, just as in the fly, and that mouse APP interacted with mouse versions of other Wnt-PCP proteins, including VanGogh and Frizzled.

This is the first study to identify a tissue-specific modulator of the Wnt-PCP pathway. Moreover, by showing that APP plays an important role in normal axonal outgrowth, the study focuses attention of whether that role is compromised in Alzheimer's disease, for instance in hippocampal neurons, whose growth is essential for new memory formation. Even if its role is not that direct, elucidating a function for normal APP may lead to new ideas for how it, or its cleavage product a-beta, causes the neuronal dysfunction and death characteristic of the disease.

**Soldano A, Okray Z, Janovska P, Tmejová K, Reynaud E, et al. (2013) The** ***Drosophila*** **Homologue of the Amyloid Precursor Protein Is a Conserved Modulator of Wnt PCP Signaling. doi:10.1371/journal.pbio.1001562**

